# Incidence of depression in people with newly diagnosed tuberculosis in Ethiopia: a cohort study

**DOI:** 10.1017/gmh.2019.27

**Published:** 2020-01-03

**Authors:** Fentie Ambaw, Rosie Mayston, Charlotte Hanlon, Atalay Alem

**Affiliations:** 1 Bahir Dar University, School of Public Health, Bahir Dar, Ethiopia; 2 Department of Psychiatry, Addis Ababa University, College of Health Sciences, School of Medicine, Addis Ababa, Ethiopia; 3 King's College London, Institute of Psychiatry, Psychology and Neuroscience, Centre for Global Mental Health, London, UK; 4 Centre for Innovative Drug Development and Therapeutic Trials for Africa (CDT-Africa), College of Health Sciences, Addis Ababa University, Addis Ababa, Ethiopia

**Keywords:** Depression, Ethiopia, incidence, primary care, tuberculosis

## Abstract

**Background:**

Cross-sectional studies show that the prevalence of comorbid depression in people with tuberculosis (TB) is high. The hypothesis that TB may lead to depression has not been well studied. Our objectives were to determine the incidence and predictors of probable depression in a prospective cohort of people with TB in primary care settings in Ethiopia.

**Methods:**

We assessed 648 people with newly diagnosed TB for probable depression using Patient Health Questionnaire, nine-item (PHQ-9) at the time of starting their anti-TB medication. We defined PHQ-9 scores 10 and above as probable depression. Participants without baseline probable depression were assessed at 2 and 6 months to measure incidence of depression. Incidence rates per 1000-person months were calculated. Predictors of incident depression were identified using Poisson regression.

**Results:**

Two hundred and ninety-nine (46.1%) of the participants did not have probable depression at baseline. Twenty-two (7.4%) and 26 (8.7%) developed depression at 2 and 6 months of follow up. The incidence rate of depression between baseline and 2 months was 73.6 (95% CI 42.8–104.3) and between baseline and 6 months was 24.2 (95% CI 14.9–33.5) per 1000 person-months respectively. Female sex (adjusted *β* = 0.22; 95% CI 0.16–0.27) was a risk factor and perceived social support (adjusted *β* = −0.14; 95% CI −0.24 to −0.03) was a protective factor for depression onset.

**Conclusion:**

There was high incidence of probable depression in people undergoing treatment for newly diagnosed TB. The persistence and incidence of depression beyond 6 months need to be studied. TB treatment guidelines should have mental health component.

## Introduction

Cross-sectional studies in both high income (Trenton and Currier, 2001) and low and middle income countries (Mathai *et al*., 1981; Aydin and Ulusahin, 2001; Moussas *et al*., 2008; Issa *et al*., 2009; Sulehri *et al*., 2010; Ige and Lasebikan, 2011; Panchal, 2011; Doherty *et al*., 2013; Duko *et al*., 2015) show that comorbid depression among people with TB is a common debilitating condition with a prevalence of as high as 50.0% (Sweetland *et al*., 2018). Recently, we have analyzed the prevalence of probable depression in people newly diagnosed with tuberculosis (TB) at the time of initiation of their anti-TB medication in primary care settings in Ethiopia and found that it was 54.0% on nine-item version of Patient Health Questionnaire (PHQ-9) at a cut-off point of 10 and above (Ambaw *et al*., 2017).

Attempts to understand the type of relationship between TB and depression are limited. A number of explanations have been given in the literature (Sweetland *et al*., 2017). Some evidence suggests that TB and depression may share risk factors (Kiecolt-Glaser and Glaser, 2002; Reiche *et al*., 2004; Katon *et al*., 2007). Others explain that TB can be contracted as a result of compromised immunity and neglected self-care associated with depression (Reiche *et al*., 2004). Such evidence shows that depression enhances the production of proinflammatory cytokines and directly minimizes immunological competence by down regulating cellular and humoral responses (Kiecolt-Glaser and Glaser, 2002; Reiche *et al*., 2004; Katon *et al*., 2007; Katon, 2011). The third hypothesis, which is also the focus of this study, is that people with TB may develop depression through various mechanisms including chronic infection and related disability and psycho-socioeconomic stressors (Mikkelsen *et al*., 2004), effects of TB medications such as isoniazid (Madan *et al*., 1989), chronic infectious conditions which may lead to overproduction of proinflammatory cytokines such as interleukin 6, which facilitate cascades of endocrine reactions that are suggested to result in depressive symptoms (Kiecolt-Glaser and Glaser, 2002), and general physical and psychological losses (Mikkelsen *et al*., 2004; Pachi *et al*., 2013). However, whether people with a newly diagnosed TB receiving anti-TB medications will develop new depressive disorder or not has not been properly studied. A study in Taiwan followed people with TB for a mean period of 6.5 years and reported that they had a higher incidence of depression compared to controls. This study has not adequately controlled socioeconomic factors and has included only cases of pulmonary TB (Yen *et al*., 2015).

Understanding whether incident depression occurs in people with TB after starting anti-TB care is critically required to improve the national guideline for clinical and programmatic management of TB in Ethiopia and similar settings that lack mental health component (Federal Ministry of Health of Ethiopia, April 2012), and to target depression interventions in the integration of mental health care into primary care which is being scaled up in Ethiopia (FMOH, 2012*a*, 2012*b*). It also provides a strong base to conduct well designed studies on the relationship between depression and anti-TB medications, specifically isoniazid (Doherty *et al*., 2013) and ethambutol (Yen *et al*., 2015), TB-related stigma or behavioral factors specific to this population (Sweetland *et al*., 2018).

The objectives of this study were to assess incidence rates of probable depression at 2 and 6 months after starting anti-TB treatment and to identify factors predicting incidence in the context of TB and its treatment.

## Methods

### Design

This study was part of a 6 month prospective observational cohort that examined the interaction between depression and newly diagnosed TB in primary care settings in Ethiopia (Ambaw *et al*., 2015). At the time of diagnosis and treatment initiation for TB, we assessed for depression and we classified participants into ‘having probable depression’ and ‘not having probable depression’ based on PHQ-9, scores. We then followed those ‘not having probable depression’ for incidence of probable depression during the 6 month follow up period.

### Study setting

The study was conducted from December 2014 to July 2016 in 14 primary care centers located in south central (i.e. in Silti and Gurage zones) and northern (i.e. Bahir Dar zone) Ethiopia. Two of the primary care centers were hospitals and 12 were health centers. Similar services were provided at the health centers and hospitals: providers of TB care were nurses or public health officers that have taken the same trainings on TB care, the same medications were given at both institutions, and the same treatment guidelines were used. All people with newly diagnosed TB in the outpatient departments were being treated according to the Directly Observed Treatment, Short course (DOTS) regimen. DOTS has 6 months duration with intensive and continuation phases. The intensive phase consists of treatment with combination of four medications (rifampicin, ethambutol, isoniazid, and pyrazinamide) for the first 2 months, and the continuation phase consists of a combination of two medications (rifampicin and isoniazid), to be taken for 4 months immediately after the intensive phase (Federal Ministry of Health of Ethiopia, April 2012).

The frontline nurses and public health officers working in those primary care centers received training in the management of mental disorders according to the evidence-based WHO Mental health Gap Action Programme Intervention Guide (mhGAP-IG) for mental, neurological, and substance use disorders in non-specialized health care settings (WHO, 2016). The presence of staff that took this training and TB patient flow of at least five per month were criteria to include the primary care centers in the study areas.

### Eligibility criteria

- People attending the selected health centers for TB treatment who were within 1 month of starting anti-TB treatment
- Aged 18 years and above
- No plan to move out of the study area
- Not too ill to be interviewed at baseline as perceived by the interviewer or the prospective participant
- Had not been admitted to an in-patient unit for more than 5 days in the last 1 month as the additional stressors of being hospitalized represent a different range of risk factors for depression.
- Not diagnosed with Multidrug-Resistant Tuberculosis (MDR-TB); people with MDR-TB constitute a different population because their treatment and outcomes are different (medications with a higher side effect burden and taken for a much longer duration; poorer prognosis) and MDR-TB is amore feared and stigmatized condition (Vega *et al*., 2004). Furthermore, only one of the study health facilities had recently started a service for people with MDR-TB.
- Not on re-treatment for TB as people who experienced previous treatment failures are at high risk of MDR-TB and constitute a different risk group for depression.

### Sample size

The total sample size was 648. The sample size was based on the primary objective of the planned longitudinal study which was to examine the effect of depression on default from anti-TB treatment (Ambaw *et al*., 2015) and was calculated using the following parameters: 80% power, 95% confidence level, 2.5% prevalence of treatment default among patients with TB and without depression, 7.5% prevalence of treatment default among people with TB and co-morbid depression and a ratio of 2:1 of non-exposed (not depressed) to exposed (depressed) participants. This provided a required sample size of 639. With a contingency of 10% for possible loss to follow up, the target sample was 703 people with TB.

### Variables and measurements

#### Dependent variable: probable depression

Depression was measured using the PHQ-9. The PHQ-9 assesses the presence of nine depressive symptoms over the past 2 weeks in an individual; the possible responses are: not at all (0), several days (1), more than half of the days (2) and almost every day (3); possible composite score ranges from zero to 27. Globally, the scale is widely used in surveys, effectiveness trials and cohort studies in various populations (Kroenke *et al*., 2010). In Ethiopia, it has been validated two times and was found to be useful in screening depression in adult out patients (Gelaye *et al*., 2013; Hanlon *et al*., 2015). The optimum cut-off point was five and above in primary healthcare centers in a rural district (Hanlon *et al*., 2015) and 10 and above in outpatient medical clinics in a referral hospital in Addis Ababa (Gelaye *et al*., 2013). We applied the more conservative cut-off point of 10 and above to define probable depression. In the baseline data of this study, the PHQ-9 had a single dimension structure, a Cronbach's α value of 0.81 and a mean inter-item correlation coefficient of 0.33 (Ambaw *et al*., 2017). In this manuscript, the term depression is used instead of probable depression for simplicity purpose.

#### Independent variables

- *Socio-demographic variables*: age, sex, marital status, level of education, religion, household income, occupation and place of residence (urban *v.* rural) were measured by self-report. Household income was measured by asking the participants to estimate the monthly total income of their household. When the participant was a farmer, we changed the estimates of annual income in kind to cash using the local market price. We converted the monthly income into annual income.
- *Perceived social support*: perceived social support was measured using the three-Item Oslo Scale of Perceived Social Support (OSLO-3) with scores ranging from 3–14 (Meltzer, 2003). The scale was previously reported to work well in TB patients in Ethiopia (Duko *et al*., 2015). In this sample the scale had an α value of 0.61 and a mean inter-item correlation of 0.35. Higher scores on OSLO-3 show better perceived social support.
- *TB-related stigma*: TB related stigma was measured at the second assessment using a 10-item TB stigma scale adapted from Macq and colleagues (Macq *et al*., 2006), translated into Amharic, and piloted (Ambaw *et al*., 2015). In this sample, the scale had an α value of 0.84 and a mean inter-item correlation coefficient of 0.34. Higher scores on the TB-related stigma scale show a higher level of stigma.
- *Substance use:* alcohol, tobacco, and khat use were measured using the WHO Alcohol, Smoking and Substance Involvement Screening Test (ASSIST) (version 3.1) (WHO, 2010). The ASSIST was designed for use across different cultural settings. The instrument's psychometric properties have been tested using data from multiple countries, including low, middle, and high income countries and shown to be valid, reliable, and easy to administer across settings (Humeniuk *et al*., 2008). The ASSIST risk score ranges from 0–31 for tobacco and 0–39 for alcohol and khat. The risk score of the respondents obtained for alcohol is categorized into ‘low’ (0 to 10), ‘moderate’ (11 to 26) or ‘high’ risk (above 26), and for khat low (0 to 3), moderate (4 to 26), and high (above 26) (WHO, 2010).
- *Comorbid illness*: data on the presence of chronic illnesses other than TB were obtained by asking the question ‘Have you ever been told by health professionals to have cardiac illness, hypertension, diabetes mellitus, depression, or mental illnesses other than depression?’ HIV status was recorded from the TB register after informed consent.
- *Type of TB*: It refers to whether the TB was pulmonary or extra-pulmonary. It was captured from the TB register in the health facilities using a structured checklist.

### Recruitment and ethics

People with newly diagnosed TB who fulfilled the inclusion criteria were identified, informed, and invited to participate in the study by health professionals running TB clinics at the health facilities. When the individuals expressed interest to participate, they were linked to trained nurse research assistants who provided them detail information, sought written informed consent or witnessed thumb print, and carried out the data collection generally at the health facilities. The information provided to participants was explained face-to-face and delivered in written form to participants. The proposal was approved by the Institutional Review Board of College of Health Sciences of Addis Ababa University (number 027/14/Psy) before data collection. In the process of data collection, respondents who endorsed the suicide item of PHQ-9 were referred to health workers within the health facilities for further evaluation and treatment.

### Follow up

**Fig. 1.**
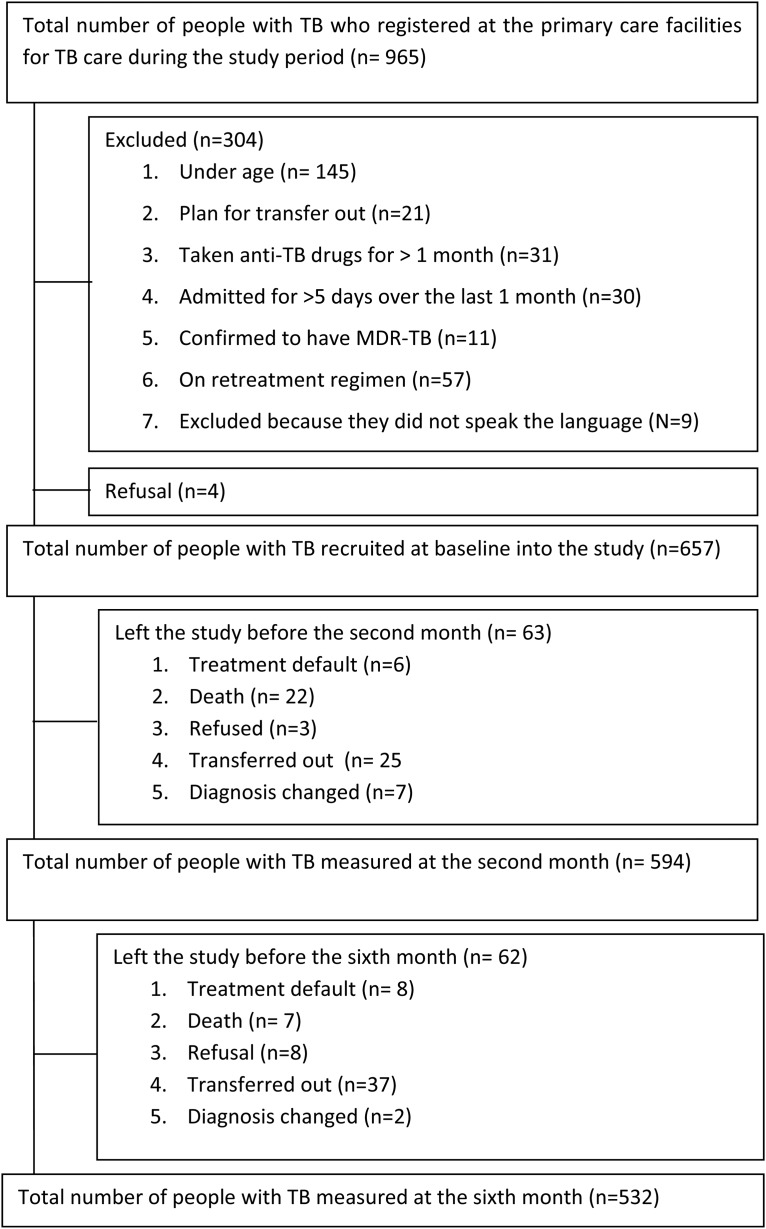
Flow chart of participants of the study.

The first (Baseline) assessment was done at the time of starting the medications. The second (2 months): assessment was done after participants took anti-TB medication for 2 months. The third (6 month) assessment was done after participants took the full course of their anti-TB medication. Figure 1 details the flow of participants at each assessment time.

### Ascertainment of time to follow up

Time of loss to follow-up was taken as midway between the last successful attempt to contact and the first unsuccessful attempt to contact, following the convention when exact times are not captured (Rothman *et al*., 2008). Participants assessed at baseline but unavailable for measurements at 2 months were defined as contributing one person-month. Participants assessed at 2 months but unavailable at 6 months were defined as contributing four person-months. Participants followed for the whole 6 months had six person-months.

### Data analysis

Data were analyzed using STATA version 15 (StataCorp. URL: http://www.stata.com). Descriptive statistics were used to summarize the data and to calculate incidence rates of depression. Poisson regression was used to identify predictors of incidence and findings were presented using adjusted *β* (beta) coefficients (Lumley and Kronmal, 2006). Robust and cluster standard errors were used as the study institutions were located in the southern and northern regions of the country. The inclusion of independent variables in the multivariable analysis was based on its theoretical importance and adequacy of the number of participants in cells for each category (Tabachnic and Fidell, 2007). Regression analysis included participants with more than one assessment for depression. Statistical significance was set at *p* value less than 0.05. The Strengthening the Reporting of Observational Studies in Epidemiology guidelines have been used to report our findings (von Elm *et al*., 2007).

## Results

### Characteristics of participants

A total of 648 participants were assessed at baseline for depression. Sixty two (9.6%) transferred out of the study area after being recruited into the study. Participants were in the age range of 18–85 years with a median of 30 years. Just over half (*n* = 348; 53.7%) were male, 224 (34.6%) had no formal education, and 172 (26.5%) were farmers. The average annual household income was 659.0 USD (standard deviation = 697.0) (Table 1). Seventy four participants (11.4%) were living with HIV. Five, three, and one participant had diabetes, cardiac illness, and hypertension respectively. No participant reported to have a previously diagnosed depression. Twelve (1.9%), eleven (1.7%), and four (0.6%) had high risk alcohol, khat, and tobacco use respectively (Table 2).

**Table 1.** Socio-demographic characteristics of participants (*n* = 648)

Variable	Number (%)/mean; range
Sex	
Male	348 (53.7)
Female	300 (46.30)
Age in years	34.4 ± 14.6; 18–85
Marital status	
Single	210 (32.4)
Married	358 (55.3)
Widowed or divorced	80 (12.4)
Level of education	
No formal education	224 (34.6)
Primary education	260 (40.1)
Secondary or above	164 (25.3)
Occupation	
Unemployed	37 (5.7)
Government employee	61 (9.4)
Self-employed	133 (20.5)
Farmer	172 (26.5)
Student	39 (6.0)
Housewife	111 (17.1)
Daily laborer	44 (6.8)
Other	51 (7.9)
Annual household income in USD	659.0 ± 697.0; 24.0–5940.0
Religion	
Christian	429 (66.2)
Muslim	219 (33.8)
Residence	
Urban	364 (56.2)
Rural	284 (43.8)
Ethnicity	
Amhara	306 (47.2)
Gurage	192 (29.6)
Mareko	68 (10.5)
Silte	65 (10.0)
Oromo	11 (1.7)
Other	6 (0.9)
Perceived social support (Oslo-3 score)	9.8 ± 2.5; 3–14
TB stigma score	27.0 ± 7.6; 10–48

**Table 2.** Illness and substance use in the participants (*N*  =  648)

Variables	Number (%)
Type of TB	
Pulmonary	371 (57.3)
Extra pulmonary	277 (42.8)
HIV status	
Negative	495 (76.4)
Positive	74 (11.4)
Unknown	79 (12.2)
Hyper tension	
No	647 (99.8)
Yes	1 (0.2)
Cardiac illness	
No	645 (99.5)
Yes	3 (0.5)
Diabetes mellitus	
No	643 (99.2)
Yes	5 (0.8)
Prior depression	
No	648 (100)
Yes	0
Level of alcohol risk:	
Low	562 (86.7)
Moderate	74 (11.4)
High	12 (1.9)
Level of tobacco risk	
Low	615 (94.9)
Moderate	29 (4.5)
High	4 (0.6)
Level of khat risk	
Low	544 (84.0)
Moderate	93 (14.3)
High	11 (1.7)

### Incidence rate of depression

Two hundred ninety nine participants without baseline depression were followed for a total of 299 and 1076 person-months at the 2nd month and the end of the follow up respectively. Two hundred seventy nine participants had more than one assessment for depression. The median duration between starting ant-TB medication and the first (baseline) assessment was zero days with a mean of 1.6 days and a standard deviation of 3.5 days. The second (2 months) assessment was done after participants took anti-TB medication for a median duration of 56.0 days with a mean of 57.0 days and a standard deviation of 3.0 days. The third (6 month) assessment was done after participants took their anti-TB medication for a median duration of 160 days with a mean of 162 days and a standard deviation of 4.8 days.

**Fig. 2.**
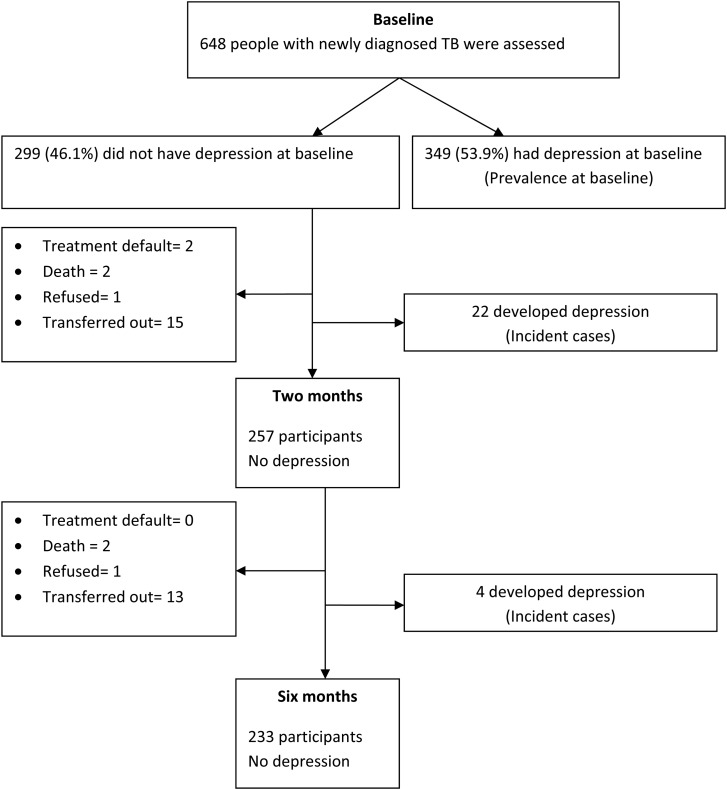
Flow chart of follow up of participants who did not have baseline probable depression.

Among the 299 participants who did not have probable depression at baseline, 22 (7.4%) developed within the first 2 months of follows up; other four participants developed probable depression in the next 4 months making the total number of new cases 26 (8.7%) (Fig. 2). The incidence rates of depression at 2 and 6 months were 73.6 (95% CI 42.8–104.3) and 24.2 (95% CI 14.9–33.5) per 1000 person-months, respectively. At 2 months, being female (adjusted *β* = 0.40; 95% CI 0.16–0.63), married (adjusted *β* = 0.37; 95% CI 0.34–0.40), and having pulmonary TB *v.* extra-pulmonary TB (adjusted *β* = 0.23; 95% CI 0.001–0.45) independently predicted incidence. Higher perceived social support was found to be protective against depression onset (adjusted *β* = −0.15; 95% CI −0.23 to −0.07). At 6 months, these variables were still independent predictors of incidence except marital status (adjusted *β* = 0.04; 95% CI −0.001 to 0.08) (Table 3).

**Table 3.** Predictors of incidence of depression in TB patients (*n* = 279)

Variables	2 months Rate (95% CI)/adjusted*β* (95% CI)	6 months Rate (95% CI)/adjusted*β* (95% CI)
Incidence rate per 1000 person-months	73.6 (42.8–104.3)	24.2 (14.9–33.5)
Sex		
Male	1	1
Female	0.40 (0.16–0.63)**	0.22 (0.16–0.27)***
Age^a^	0.01 (−0.01–0.02)	0.001(−0.001–0.02)
Marital status		
Single	1	1
Married	0.37 (0.34–0.40)***	0.04 (−0.001–0.08)
Widowed or divorced	0.56 (−0.27–1.38)	0.12 (−0.84–0.08)
Level of education		
No formal education	1	1
Primary education	1.10 (−0.15 to 2.34)	0.71 (−0.63 to 2.06)
⩾ Secondary	−1.10 (−3.99 to 1.79)	−1.59 (−4.85 to 1.66)
Religion		
Christian	1	1
Muslim	0.23 (−0.39 to 0.85)	0.15 (−0.86 to 1.17)
Residence		
Urban	1	1
Rural	0.17 (−0.06 to 0.40)	0.08 (−0.27 to 0.43)
Annual household income^a^	0.001 (−0.001 to 0.001)	−0.001 (0.001–0.001)
Perceived social support^a^	−0.15 (−0.23 to −0.07)***	−0.14 (−0.24 to −0.03)**
TB related stigma^a^	0.10 (−0.03 to 0.24)	0.09 (−0.03 to 0.21)
HIV status		
Negative	1	1
Positive	−0.69 (−3.62 to 2.23)	−0.73 (−3.28 to 0.82)
Unknown	−1.09 (−1.28 to −0.90)	−0.01 (−0.33 to 0.32)
Type of TB		
Pulmonary	0.23 (0.001–0.45)*	0.29 (0.18–0.40)***
Extra pulmonary	1	1

Adjusted *β* = regression coefficients;* = *p* < 0.05; ** = *p* < 0.01; **** = *p* < 0.001

Adjustment was done for all the variables in the table.

a The variables were measured at the continuous level.

## Discussion

A high incidence rate of probable depression was found in people with newly diagnosed TB undergoing treatment using the DOTS regimen. In a previous manuscript we found that depression was associated with increased mortality and treatment default rates in people with TB (Ambaw *et al*., 2018). In general, depression co-occurring with physical conditions decreases treatment adherence (Katon, 2011) and substantially increases mortality (Wulsin *et al*., 1999). Taking the harm comorbid depression in people with TB can cause (Sweetland *et al*., 2017; Ambaw *et al*., 2018), and the fact that the incident cases occurred in people who were under the attention of healthcare providers into consideration, the incidence rate observed in our study can be interpreted as alarmingly high. In fact, the incidence rate we found is higher than what was reported for people living with HIV both in Uganda, Africa (Kinyanda *et al*., 2016) and France, Europe (Nacher *et al*., 2010; Elenga *et al*., 2014), type-2 diabetic patients (Lunghi *et al*., 2016), or nursing homes (Boorsma *et al*., 2012). Previous researchers also reported that depression is more prevalent in people with TB than with other physical illnesses (Doherty *et al*., 2013). The global incidence rate of unipolar depression was estimated to be 49/100000/year for women and 31/100000/year for men in 2000 (Ustun *et al*., 2004), incomparably lower than that observed in TB suggesting that TB care guidelines should include mental health components. Sweetland and colleagues (Sweetland *et al*., 2018) have proposed that TB care is an appropriate component of the existing health services to integrate mental health in primary care in low-resource settings.

Higher incidence of depression in people with TB compared to the general population had been reported in Taiwan (Shen *et al*., 2014, Yen *et al*., 2015). The incidence rate observed in our study was substantially higher than that reported in Taiwan probably because studies in Taiwan followed people with TB for about 12 years including mainly TB-free period of the participants (Shen *et al*., 2014; Yen *et al*., 2015). Shen *et al*. (2014) found the highest number of incident cases during the first 6 months which seems to agree with our finding.

New onset depression in people with newly diagnosed TB could be the result of the active inflammatory process directly leading to depression in susceptible individuals (Herbert and Cohen, 1993; Raison *et al*., 2006; Dantzer *et al*., 2008), stress of encountering an infectious diseases with risk to others, decreased functioning from illness and psychological loss (Pachi *et al*., 2013), and, taking multiple drugs with warnings that failure to adhere will have dire consequences. The first 2 months from the start of anti-TB medications is also a period when most medication side effects occur (Yee *et al*., 2003; Gulbay *et al*., 2006). With time, people with TB are more likely to develop better coping strategies (Olley *et al*., 2006). The relative reduction in the incidence rate after 2 months in the presence of continued isoniazid and discontinued ethambutol doses may be a point of interest for future investigation on the relationship of these medications with depression.

Our finding that female sex predicts incidence of depression agrees with previous studies in various populations (Kessler *et al*., 1993; Buchtemann *et al*., 2012; Huang *et al*., 2012; Nefs *et al*., 2012; Elenga *et al*., 2014; Lunghi *et al*., 2016). Likewise, the protective effect of social support was in agreement with what was observed in diabetes cohort (Lunghi *et al*., 2016). This finding can be a supportive evidence to assert the notion that enhancing the support system of TB patients can reduce depression. The higher incidence of depression in people with pulmonary TB than for extra pulmonary TB may be related to the higher communicability of pulmonary TB which puts all close contacts, mainly household members, at a high risk of developing TB, and this undue cognitive stress could lead to depression; but biological mechanisms should also be investigated.

## Limitations

We used a screening tool to assess depression. We might have reported undiagnosed prevalent cases as incident as the health system may not be strong enough to diagnose depression in the context of TB where there is overlap between TB and depression symptoms. For example, none of the participants who scored above the cut point of PHQ-9 and none of the participants who we referred to health professionals because they endorsed the ‘suicide item of PHQ-9’ were diagnosed to have depression by the trained primary care workers.

Undiagnosed physical illnesses, poverty, and loss to follow up might have confounded our findings. Because the study was conducted in a setting where the health system is not as such strong, our participants could have had undiagnosed comorbid physical illnesses that might have increased incidence of depression. Another potentially confounding factor is poverty which may not have been fully captured by our socio-demographic variables. Lastly, we had no information on whether participants transferred out of the study area differed significantly from others in terms of developing depression. In addition to that our conclusions cannot be extended to TB patients who are hospitalized, are being re-treated, or have multidrug-resistant disease.

Our sampling technique was a family of non-probability sampling. Nevertheless, as the consecutive sample met criteria for robustness (multiple sites, long data collection period, all eligible participants approached to participate) (Mathieson, 2014) it should be reasonably representative of adults with newly diagnosed TB in the outpatient department. Theoretically, information bias was a possibility due to a change in the capacity of the assessors as they become more familiar with more practice over time. There are no statistical methods to check this bias and its direction cannot be determined in this specific situation. However, the rigorous training given to field workers before starting data collection is expected to minimize this bias.

A potential concern regarding generalizability is homogeneity of the TB treatment approach in other settings and the sustainability of the current practice of TB treatment in future (Partridge *et al*., 2015). TB is being treated according to the DOTS approach introduced by the WHO in 1994 (Onozaki and Raviglione, 2010) and this approach is being adhered in the global strategy of ‘End-TB by 2030’ (World Health Organization, 2015). It is therefore similar across low-income country settings and is likely to be sustained in the coming years. In conclusion, the observed findings would seem to be reasonably valid for the study population as well as being generalizable to adults with newly diagnosed TB in outpatient settings elsewhere in Ethiopia as well as in similar settings in Sub Saharan Africa.

## Conclusions

The high incidence rate of probable depression was found in people with newly diagnosed TB and undergoing treatment. Most incident cases occurred during the first 2 months and the incidence rate relatively decreased with time during the continuation phase of anti-TB treatment despite continued use of isoniazid. The long term course of incident cases of depression, and for how long incidence rate remains high in this population after the TB is treated requires further study. TB care guidelines should have mental health components.
